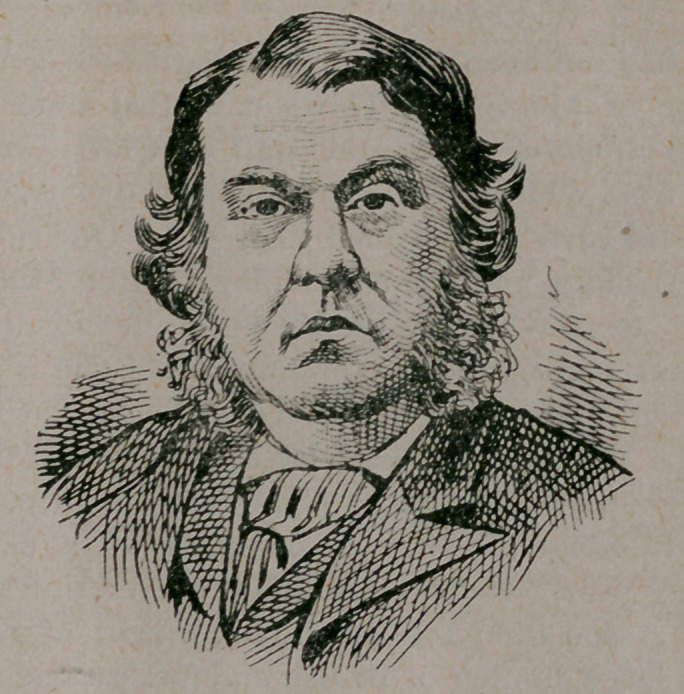# Lawson Tait, F. R. C. S.

**Published:** 1887-04

**Authors:** 


### Southern Medical Record.

A MONTHLY JOURNAL OF PRACTICAL MEDICINE.

☞All Communications must be addressed to R. C. WORD, M. D., Managing Editor.

"*Quicquid Præcipies Esto Brevis.*"

VOL. XVII. ATLANTA, GA., APRIL, 1887. NO. 4.

### ORIGINAL AND SELECTED ARTICLES.

LAWSON TAIT, F.R.C.S.

Mr. Tait, the subject of the present sketch and illustration (says the *Provincial Medical Journal*), was born in Edinburgh in the year 1845. He is the only surviving son of Archibald Campbell Tait, a Guild Brother of the well known Heriot's Hospital, into which Lawson Tait was admitted at the age of seven. He had a distinguished career in the school, and was sent on a scholarship to the University of Edinburgh, where he passed through the cirriculum of Arts, and afterwards of Medicine. During his medical studies—from 1860 to 1866—he was under the immediate guidance of a young operating surgeon of great promise, the late Alexander McKenzie Edwards, the favorite pupil of the late Sir William Ferguson, and he was also closely associated with the late Sir James Young Simpson, to whose line of practice he has since exclusively devoted himself. Mr. Tait originally intended to remain in practice in his native town, but this intention was altered owing to the death of his master, and after a brief sojourn in Wakefield, as house-surgeon to the local hospital, he selected Burmingham as a field where an opening for his special work presented itself. In this town he settled in September, 1870, taking up his professional quarters in the house of Dr. Bell Fletcher. Shortly after settling, he connected himself with the movement to establish a hospital for the special diseases of women in Birmingham, and with this Institution he has been prominently associated ever since its foundation. In 1871 he married Sibyl Anne, daughter of William Stewart, a solicitor in Wakefield.

Mr. Tait had not been long in Birmingham when he made the acquaintance of Mr. George Dawson, at that time editor of the Birmingham *Morning News*, the staff of which journal Mr. Tait joined, and from which connection he derived considerable advantage during his early professional struggles. Our subject was lecturer on physiology and general biology at the Midland Institute from 1871 to 1879, when his increasing professional engagements compelled him to resign these appointments. He is at the present time professor of anatomy to the Royal Society of Artists and to the School of Design. He was elected a Fellow of the Royal College of Surgeons of Edinburgh (honorary) in 1870, and a Fellow of the Royal College of Surgeons of England (by examination) in the following year. He is a prominent member of the Council of the British Association for the Advancement of Science, and a Fellow of a large number of British and foreign scientific societies. He is chairman of the Birmingham Health Committee, president of the Birmingham Philosophical Society, and chairman of Clinical Section of branch of British Medical Association. Mr. Tait has always taken a lively interest in the public and municipal life of the town in which he lives, scientific, literary and political, and his attention has been especially directed to its sanitary condition, and to all subjects connected with the health of the people.

In 1866 he was elected for the Bordesley Ward in the Town Council, and two years afterwards, on his election being opposed, he was again returned by a large majority. He is a member of the Health and Asylum Committees, on which his large knowledge and eminent ability have been of the greatest service. Mr. Tait was the founder of the Midland Union of Natural History Societies, and of the *Midland Naturalist*, and the promoter of the Birmingham Provident Dispensaries. He was also largely concerned in the establishment of "coffee-houses" in Birmingham. As an author and contributor to medical literature he is well and widely known, the contributions from his pen being too numerous for detail mention in the space now at our command. His recent address delivered before the Birmingham Medical Society on "A Series of One Thousand Cases of Abdominal Section" will be fresh in the recollection of our readers. Upon the subject of this address the *Medical Press*, of February last, had a leading article of a most eulogistic character, which concludes as follows: "No one will withhold from Mr. Tait the hearty congratulations to which he is entitled on the publication of his experience of one thousand cases of abdominal section. No one either, we venture to think, will be found to dissent from the opinion that such a record as this publication contains is one of which any surgeon in the world might well be proud."

The first abdominal section performed by our subject was in the year 1867, and the first important contribution which he made on the subject was his essay on "Diseases of the Ovaries," which gained for him the Hastings Gold Medal at the meeting of the British Medical Association, in London, in 1873. The presentation was made by Sir William Ferguson, who said he had "to congratulate Mr. Tait upon obtaining the distinction, and this honor was the greater inasmuch as the award was not made as a matter of course, for though in some years there had been competing essays of singular merit sent in, the medal had not always been given, while this year it had been awarded with every imaginable approbation, and that circumstance showed the winning essay very much redounded to the credit of the writer. The question proposed was one of modern date, and one on which some of the best writers of the day had been engaged. This being the fact, it was marvellous to him that the committee of these essays should have decided that Mr. Tait had achieved the distinction which entitled him to the medal. The subject was one of the most original surgical subjects of the century, for it was one which had been developed in modern surgery. It was something for Mr. Tait to have earned distinction in this way, and all present would feel themselves honored in having the opportunity of congratulating Mr. Tait. It was his duty to congratulate Mr. Tait in the name of the association, and he performed the task with the greatest pleasure. He proceeded to refer to the teaching of the Medical School of Edinburgh, of which Mr. Tait had proved himself a distinguished pupil ; but the winning of this medal had been his greatest distinction, and he had done honor to the teaching of Sir James Simpson, who had done so much for modern surgery and medicine. He concluded by wishing Mr. Tait prosperity in a career which he had so admirably begun." Until 1877 and 1878 the School of Abdominal Surgery, originated at Birmingham by Mr. Tait, made no satisfactory progress, but at that time his attention was drawn to the remarkable results in ovariotomy Keith obtained. chiefly by the revival of the principle of intra-peritoneal treatment of the pedicle as originally devised by Nathan Smith and practiced by Baker Brown. The brilliant results thereby obtained were so satisfactorily equalled in Mr. Tait's own practice that he was enabled to reduce his ovariotomy mortality from 30 down to 3 per cent. At this time the doctrines of Lister were obtaining a large hold on the professional mind, and they were applied by Keith to the performance of abdominal operations. Mr. Tait on first principles did not accept the Listerian doctrines, but he gave a long and careful trial to the details of the system ; a trial which, having extended over three years, induced him finally and completely to abandon Listerism, and his recently published statistics of 313 cases with a mortality of under 5 per cent. have completely justified his action. As a natural result of his increasing success in the removal of ovarian tumors he extended his field of work and began to deal with a number of diseases, many of which, up to that time, were either entirely unrecognized or not considered to be within the limits of the art of surgery. In 1872 Mr. Tait first suggested, simultaneously with Hegar, and carried into effect with complete success, the removal of the uterine appendages for the treatment of myoma, but the results of this practice in his early work were such as to deter him from extending it to any very great extent. As soon, however, as the statistics of ovariotomy came to be so low, he at once resumed his extension of abdominal surgery and engaged in the performance of large numbers of new operations which have excited a very great deal of discussion and some adverse criticism. The main points upon which opposition was levelled were the removal of the uterine appendages for chronic inflammatory disease, but demonstration after demonstration, both of operations performed and diseased appendages removed, made it perfectly clear that the proceeding had been entirely misunderstood, and that the charges which had been made of removing normal ovaries could not be in any way maintained, so that now the operations have been completely vindicated, and their practice is being followed in every country in Europe and throughout the American continent. Large numbers of foreign visitors have flocked to Birmingham to see these operations and have carried away with them specimens of the disease, which are now exhibited in the chief medical museums of the world. In addition to these special advances, Mr. Tait also introduced a new method of dealing with that most untractable disease—pelvic abscess—by the perfectly safe and speedy method of opening the abdomen and draining the abscess from above. Thirty of these operations have been performed without a death and with complete cure of the disease. Mr. Tait was also the first to perform, successfully, the operation originally devised by Dr. Marion Sims, of opening the gall bladder for the removal of the stones. This operation he has performed thirteen times, in every case successfully. He also extended this proceeding in the operation of hepatotomy, actually cutting into the substance of the liver and removing thence large hydatid tumors, these having been completed ten times, in every instance the performance being a perfect cure of the disease. Another advance of a material kind in abdominal surgery is due to the inovation advocated and introduced by Mr. Tait, of treating cases of obstruction of the bowels by opening the abdomen and establishing an artificial anus on the first presenting piece of intestine which is distended, instead of, as was formerly practised, hunting about for an obstruction, which was very rarely found. This proceeding he has performed eight times, with only one death. Similarly, he has advocated the practice of opening the abdomen in chronic and acute peritonitis, cleaning the cavity and draining it, and here his new practice has again been followed by the most marvellous success. The extremely fatal cases of Fallopian pregnancy rupturing in the early months and causing death by hemorrhage, have been cured by him five times out of six, another additional success to these novel and daring procedings, whilst his published records teem with cases of successful treatments in tumor of the kidney, spleen, and uterus. He has also advanced wholly new views on the pathology of extra-uterine pregnancy and other points which have met with general acceptance.

## Figures and Tables

**Figure f1:**